# Sex-specific covariance between metabolic rate, behaviour and morphology in the ground beetle *Carabus hortensis*

**DOI:** 10.7717/peerj.12455

**Published:** 2021-12-15

**Authors:** Elisabeth Yarwood, Claudia Drees, Jeremy E. Niven, Wiebke Schuett

**Affiliations:** 1 School of Life Sciences, University of Sussex, Brighton, East Sussex, United Kingdom; 2 Institute of Zoology, Universität Hamburg, Hamburg, Germany

**Keywords:** Body size, Body mass, Carabid, Metabolism, Personality, Sex difference, Exploration, Novel environment

## Abstract

**Background:**

Individuals within the same species often differ in their metabolic rates, which may covary with behavioural traits (such as exploration), that are consistent across time and/or contexts, and morphological traits. Yet, despite the frequent occurrence of sexual dimorphisms in morphology and behaviour, few studies have assessed whether and how sexes differ in metabolic trait covariances.

**Methods:**

We investigated sex-specific relationships among resting or active metabolic rate (RMR and AMR, respectively) with exploratory behaviour, measured independently of metabolic rate in a novel environment, body size and body mass, in *Carabus hortensis* ground beetles.

**Results:**

RMR, AMR and exploratory behaviour were repeatable among individuals across time, except for male RMR which was unrepeatable. Female RMR neither correlated with exploratory behaviour nor body size/body mass. In contrast, AMR was correlated with both body size and exploratory behaviour. Males with larger body sizes had higher AMR, whereas females with larger body sizes had lower AMR. Both male and female AMR were significantly related to exploratory behaviour, though the relationships between AMR and exploration were body mass-dependent in males and temperature-dependent in females.

**Discussion:**

Differences between sexes exist in the covariances between metabolic rate, body size and exploratory behaviour. This suggests that selection acts differently on males and females to produce these trait covariances with potentially important consequences for individual fitness.

## Introduction

Individuals within a species often display consistent differences in metabolic rate (Nespolo & Franco, 2007; Biro & Stamps, 2010; Burton et al., 2011). Metabolic rate is an important element of life-history that exists in a trade-off with growth, reproduction and survival (Stearns, 1992; Burton et al., 2011). Understanding the processes that produce individual differences in metabolism are therefore important because intraspecific differences in metabolism may influence individual fitness. Intraspecific variation in metabolic rate may also have significant impacts at the population level by influencing individual reproductive rates and survival (Burton et al., 2011).

Intraspecific variation in metabolic rate may be associated with traits such as body mass (*e.g.*, Killen, Atkinson & Glazier, 2010; reviewed in: Glazier, 2005), or linked to distinct ‘personalities’ (Careau et al., 2008). In this context, ‘personality’ refers to consistent individual differences in a behavioural trait across time (and/or context) (*e.g.*, Dall, Houston & McNamara, 2004; Réale et al., 2007; Bell, 2007; Schuett, Tregenza & Dall, 2010; Stamps & Groothuis, 2010; reviewed in: Sanchez-Tojar, Moiron & Niemelä, 2021).

Three different hypotheses attempt to explain the relationship between resting metabolic rate (RMR) and personality differences: (1) the ‘performance hypothesis’ (positive relationship between RMR and personality: high RMR drive behaviours that feed-back high energy input); (2) the ‘allocation hypothesis’ (negative relationship between RMR and personality: energy is a finite resource that is split between the two) (Careau et al., 2008), and; (3) the ‘independent hypothesis of energy management’ (no relationship) (Careau & Garland, 2012). Each of these three hypotheses are supported by evidence from the literature. Some studies support the performance hypothesis (*e.g.*, Careau et al., 2011; Videlier, Rundle & Careau, 2019), whilst others observed a negative correlation between personality and RMR, thereby supporting the allocation hypothesis (*e.g.*, Bouwhuis et al., 2014; Biro et al., 2020). Still, other studies support the independent hypothesis of energy management in that they found no significant relationship between personality and RMR (*e.g.*, Bouwhuis et al., 2014; Videlier, Rundle & Careau, 2019; Agnani et al., 2020). Consequently, the relationships between intraspecific RMR, body mass/body size and animal personality traits remain largely unclear. Even less clear are the potential associations between active metabolic rate (AMR), body mass/body size and animal personality traits. Nevertheless, intraspecific differences in AMR may be more tightly linked to independently measured personality differences than RMR, because the energetic cost of movement should influence whether an individual engages in more or less energy expending behaviours. Studying the links between AMR and personality traits is important because intraspecific differences in energy expenditure during movement may affect the energy available for growth, somatic maintenance, and reproduction.

Despite the increasing interest surrounding the relationships between metabolism, personality, and morphology, the majority of studies investigating metabolic trait covariances neglect one important factor: sex (Hämäläinen et al., 2018). This is particularly surprising given sex differences are often considered in studies investigating solely RMR (*e.g.*, Hill, Silcocks & Andrew, 2020), personality traits (*e.g.*, Schuett & Dall, 2009), or morphology (*e.g.*, Yarwood et al., 2021). Differences in reproductive strategies and investment between the sexes arise as consequences of anisogamy (Bateman, 1948; Maynard Smith, 1978), which may lead to differences in traits associated with reproduction (Bateman, 1948), and/or differences in the fitness benefits of investing in metabolic rate, personality traits and morphological traits (Hämäläinen et al., 2018). The latter case may lead to sex-specific trait-covariances, with the strength (and potentially direction) of correlations between traits differing between the sexes because the trait-covariance is more beneficial to one sex than the other (Hämäläinen et al., 2018). Furthermore, the majority of studies investigating relationships between metabolic rate, and both personality traits and morphological traits have focused on endothermic vertebrates, largely ignoring insects (but see: Royauté et al., 2015). This is despite clear differences in the physiology and morphology of insects in comparison to endothermic vertebrates (Schmidt-Nielsen, 2007), that might affect metabolic trait covariances (*e.g.*, Mathot, Dingemanse & Nakagawa, 2019).

Here we investigate the relationships between metabolic rate (RMR and AMR), exploratory behaviour in a novel environment, body size and body mass in both males and females of the predatory, nocturnal ground beetle *Carabus hortensis* Linnaeus, 1758. *C. hortensis* are flightless (Turin et al., 2003), obviating the need to measure flight metabolic rate to obtain active metabolic rate measures. Moreover, in other closely-related flightless ground beetle species, individual exploratory behaviour has been found to: (a) be repeatable across individuals over time, meaning that individuals display personality differences in exploration; and (b) positively correlate with another behavioural trait: risk taking (Schuett et al., 2018). The body size of male, but not female *C. hortensis* has been shown to increase towards range edges, with which the male to female sex ratios also increased. Body size may therefore be more important to male than female reproductive success (Yarwood et al., 2021). Furthermore, males of closely-related carabid beetles show higher locomotory activity than females (*e.g.*, Drees & Huk, 2000; Gerlach, Voigtländer & Heidger, 2009; Lagisz et al., 2010), which likely serves to increase the rate at which individuals encounter potential mating partners (Drees & Huk, 2000). If *C. hortensis* show similar sex-specific activity patterns, then males may expend more energy and have greater fitness benefits associated with exploration than females. These sex-differences in behaviour and the selection-pressures upon morphological traits may cause sex-differences in *C. hortensis* average trait values and in the direction and slope of trait covariances.

We measured metabolic rate and exploratory behaviour independently of one another to reduce the possibility that correlations between them are caused by immediate influences of one on the other. Such correlations could occur regardless of whether individuals consistently differ in behaviour and metabolism and hence could produce erroneous conclusions. We measured the repeatability of RMR, AMR, and exploratory behaviour in a novel environment over time across individuals, assessing the presence of consistent intraspecific differences in metabolism and personality. We first analysed the relationships between metabolic rates, exploratory behaviour, body mass and body size with both male and female combined data to assess whether the sexes differ in their average trait values. We then measured the relationships between traits using separate male and female data to determine sex-specific metabolic trait covariances.

We hypothesise that: (1) *C. hortensis* individuals show consistent personality differences in exploratory behaviour and consistent differences in metabolic rates; (2) *C. hortensis* metabolic rate scales with body size/body mass; (3) RMR and AMR are positively (‘performance hypothesis’) or negatively (‘allocation hypothesis’) correlated with exploratory behaviour (Careau et al., 2008); (4) if the relationship between metabolic rate and exploratory behaviour is positive, then average RMR and AMR may be higher in males than females; (5) if the relationship between metabolic rate and exploratory behaviour is negative, then females may have higher average RMR and AMR than males; and (6) the relationships between metabolic rate, exploratory behaviour, body size, and body mass are stronger in males than in females.

## Materials & Methods

### Study species, trapping and maintenance

*Carabus hortensis* (Coleoptera, Carabidae) Linnaeus, 1758, ground beetles were collected from the Lüneburger Heide, Lower Saxony, Germany (N53°10′53.32″, E9°53′08.06″) (Yarwood et al., 2021). In total, 62 females and 26 males were caught between August-September 2018 during the reproductive season of the beetles (Günther & Assmann, 2000), using live pitfall traps (Schuett et al., 2018; Yarwood et al., 2021). Traps were baited with cellulose soaked in red wine and were emptied/re-baited every 7–8 days (*e.g.*, Ernst & Buddle, 2013; Marcus et al., 2015; Schuett et al., 2018). Collected individuals were housed separately in 10 (L) × 7.5 (W) × 4.5 (H) cm containers containing peat, and regularly sprayed with water to ensure a moist environment. Beetles were fed mealworm (*Tenebrio molitor*) pupae *ad libitum*. The light and temperature at which individuals were stored was reduced in increments over time, once per week, to mimic daylight and temperature changes in the natural environment, thereby promoting natural behaviours and metabolic rates. The experiment lasted from October 2018 to February 2019 during which time the conditions in which the beetles were kept changed from a 12:12 h 13.8:6.6 °C light-dark regime to an 8.5:15.5 h light-dark regime at 5.8 °C. Prior to making behavioural and metabolic measurements, beetles were starved for 2 days to ensure that they were in a post-absorptive state.

### Behavioural tests

All behavioural tests were conducted immediately before all metabolic measures. To measure individual exploratory behaviour, individuals were placed at the centre of an open white 37.5 (L) × 26 (W) cm plastic box with a 28 × square grid on the base (Schuett et al., 2018). The number of squares visited, including repeated visits to the same square, were counted during observation for 90 s to assess individual exploratory behaviour. Temperature was recorded once every 10 min throughout behavioural trials using data loggers (Voltcraft DL-210TH; Conrad Electronic SE, Hirschau, Germany) and ranged from 11.5–24.1 °C (17.3 ± 2.3 °C mean ± SD). Two measurements of exploratory behaviour were taken 13–15 days apart to assess whether differences among individuals were consistent over time.

### Measuring metabolic rate

A L1-7000 dual channel CO_2_ infra-red gas analyser (LI-COR, Lincoln, NE, USA), operating in differential mode at 2 Hz with two identical chambers was used to measure individual *C. hortensis* metabolic rates (Perl & Niven, 2018). One chamber was empty acting as a reference chamber whilst the other chamber contained the beetle, allowing a differential measurement of CO_2_. Chambers were 115 (L) × 30 (W) mm, with a 50 ml capacity, allowing ample space for beetle movement. Air was pumped into the chambers using a SS4 Subsampler (Sable Systems International, Las Vegas, NV, USA) through soda lime and Drierite (W.A. Hammond Drierite, Xenia, OH, USA) scrubbing columns, to remove CO_2_ and H_2_O, respectively, before it was split between two mass flow controllers (GFC17; Aalborg, New York, NY, USA) that maintained airflow into two chambers at 100 ml min^−1^. Temperature was recorded once every 10 min using Voltcraft DL-210TH data loggers (Conrad Electronic SE; Hirschau, Germany), and ranged from 14.4–23.3 °C (mean = 18.1 ± 2.0 °C SD). Temperatures at which metabolic measurements and behavioural measurements were taken differed because metabolic and behavioural measurements were conducted in different rooms. Individuals were allowed to move freely throughout metabolic measurements. We filmed the metabolic rate chamber with a high-speed camera (JVC GC-PX100; JVC Ltd, Yokohama, Tokyo, Japan) operating at 72 frames per second to classify periods when beetles were stationary and when they were moving.

RMR measures were conducted over 30 min between 08:00 and 16:00, immediately after assessing individual exploratory behaviour. An LED work light (Sealey WL483D 230V, Sealey Tools, Bury St Edmunds, UK) was used to replicate daylight. AMR was measured over 12 h and took place during the night (20:00–08:00), during the *C. hortensis* active period, after assessing individual exploratory behaviour. AMR trials took place over 12 h rather than 30 min due to difficulties with conducting multiple 30-min metabolic measurements throughout the night. A red lamp was used for illumination: ground beetles cannot see the wavelengths of red light (*e.g.*, Hasselmann, 1962; Drees, Matern & Assmann, 2008). All 88 beetles were tested twice during the day (with 13–15 days between repeated trials) to assess repeatability of RMR over time; 43 of the 88 beetles (25 females and 18 males) were also tested once for their AMR and behaviour overnight, so that these 43 individuals were tested for their metabolic rate three times (with RMR measured twice and AMR measured once). The night-time AMR of the remaining 45 individuals was not measured.

### Metabolic rate analysis

Videos of the beetles within chambers were analysed offline using JWatcher software (version 0.9) (Blumstein & Daniel, 2007). Measurements of RMR were made only when beetles were stationary during daytime metabolic measurements. Conversely, AMR estimates were obtained from periods when beetles were active during the night-time metabolic measurements. Estimates of RMR and AMR in CO_2_ μl min^−1^ production were calculated using Origin(Pro) 2016 (OriginLab Corporation, Northampton, MA, USA) software, from time periods when individuals were at rest or active, respectively. RMR was measured when beetles were at rest for 3 min or longer and was estimated from the last minute of inactivity. RMR was averaged across all periods of inactivity within a single 30-min trial. AMR was averaged across all periods of activity within a single 12-h trial. Volumes of CO_2_ μl min^−1^ for separate periods of activity and rest were converted to provide the total volume produced per hour.

*C. hortensis* beetles performed three different types of ventilation: continuous, discontinuous, and pulsatile (Gudowska et al., 2017b). Ventilation patterns produced by beetles were visually classified in Origin(Pro) 2016. Traces were classified as continuous respiration where we visually observed that CO_2_ output was continuous, and troughs did not reach 0 μl min^−1^. We classified respiration patterns as discontinuous when we visually observed multiple cycles within a 30-min time period of CO_2_ μl min^−1^ decreasing sharply to and plateauing at 0 μl min^−1^ for 100 s or longer, before sharply increasing. We classified respiration patterns as pulsatile when we visually observed rise and falls in CO_2_ μl min^−1^ similar to discontinuous respiration, but in which: (a) the length of time over which CO_2_ μl min^−1^ plateaued at 0 μl min^−1^ was almost equal to the time where CO_2_ μl min^−1^ was above 0 μl min^−1^; and (b) where there were obvious, individual peaks of CO_2_ output. Although some studies have shown that ventilation pattern has no significant effect on metabolic rate scaling (*e.g.*, Gudowska et al., 2017a), others have shown that metabolic rate can scale differently with body mass when CO_2_ production from continuous, discontinuous and pulsatile ventilation patterns are analysed together *versus* separately (*e.g.*, Perl & Niven, 2018). We therefore measured the trait covariances of CO_2_ production values from continuous ventilation patterns separately from those of CO_2_ production values from discontinuous and pulsatile ventilation patterns. Due to the small number of instances in which beetles performed discontinuous or pulsatile respiration (15 RMR traces, seven AMR traces), these breathing patterns were excluded from analysis.

Sample sizes available for different analyses differed. Twenty-one females and nine males were excluded from RMR analyses because they either: (a) remained active throughout both RMR trials (14 females, nine males); (b) performed discontinuous or pulsatile respiration throughout both RMR trials (two females); (c) remained active throughout one RMR trial and performed discontinuous or pulsatile respiration throughout the other RMR trial (three females); or (d) remained active throughout one RMR trial and died before a second could be taken (two females). RMR analyses were, therefore, conducted on 58 individuals (41 females, 17 males).

Nine females and two males were excluded from AMR analyses because they either: (a) remained inactive throughout the AMR trial (two females); (b) performed discontinuous or pulsatile respiration (five females, two males); or (c) died shortly afterward (two females). AMR analyses were therefore conducted on 32 individuals (16 females, 16 males).

### Measurements of body mass and pronotum width

We measured both body mass and pronotum width as a proxy for body size (Yarwood et al., 2021) to investigate the relationships between body size/body mass and metabolic rate. Dorsal photos were taken of each individual over a laminated page of mm grid paper using a Wileyfox Swift 2× camera phone (Wileyfox, London, UK). ImageJ (version 1.53K) (Schneider, Rasband & Eliceiri, 2012) was used to measure the widest section of the pronotum to the nearest 0.1 mm. To account for changes in body mass during metabolic measurements, beetles were weighed (Precisa 125A; Precisa Limited, Livingston, UK) to the nearest milligram, immediately before and afterward. These two weight measurements were then averaged to provide a measure for average body mass for the duration of the metabolic measurement.

### Statistical analysis

All statistical analyses were carried out in R version 3.3.2 (R Core Team, 2019).

### Consistency of exploratory behaviour and metabolic rates over time

Linear mixed effects models (LMMs) were used with the rptR package (Stoffel, Nakagawa & Schielzeth, 2017) to estimate repeatability of RMR, AMR and exploratory behaviour for combined male and female data as well as separately for each sex. For AMR, repeatability estimates were obtained from samples 4−8 h apart: the first from 0−2 h from the start of metabolic testing and the second from 6–10 h. To account for differences in the temperature at which metabolic rate and behavioural trials were conducted between repeated tests, temperature was included as a covariate in all cases, thus adjusting repeatability. Beetle identity (‘ID’) was included as a random term. Confidence intervals of 95% were used to infer the significance of the repeatability of exploratory behaviour and metabolic rates; if the confidence interval included zero, the trait was considered not repeatable.

Male RMR was not repeatable over time, however, the sample size was considerably smaller than that of female RMR (Table 1). To assess whether a small small sample size may have affected male RMR repeatability, we performed 1,000 permutations of repeatability on subsets of female data, where the subset size equalled the total male sample size (*i.e.*, 17 individuals). From these tests, we determined that female RMR was repeatable in only 43% of cases in which the sample size was 17, suggesting that low sample size may explain why male RMR was unrepeatable.

**Table 1:** Repeatability estimates (±95% confidence intervals) from linear mixed effects models for active metabolic rate (AMR), resting metabolic rate (RMR) and exploratory behaviour.

Response variable	Dataset	Mean Temp ± 1SD	Repeatability	95% CI	n_ID_ (n_Obs_)
AMR	M + F	21.5 ± 0.8	**0.644**	**[0.332–0.856]**	32 (50)
	F	21.5 ± 0.9	**0.696**	**[0.324–0.902]**	16 (29)
	M	21.5 ± 0.7	**0.698**	**[0.017–0.960]**	16 (21)
RMR	M + F	17.5 ± 1.7	**0.419**	**[0.043–0.709]**	58 (80)
	F	17.6 ± 1.9	**0.524**	**[0.055–0.830]**	41 (55)
	M	17.4 ± 1.2	0.111	[0.000–0.784]	17 (25)
Exploration	M + F	17.3 ± 2.3	**0.367**	**[0.169–0.544]**	88 (171)
	F	17.4 ± 2.4	**0.247**	**[0.009–0.478]**	62 (119)
	M	17.1 ± 2.1	**0.484**	**[0.123–0.738]**	26 (52)

**Note:**

Repeatability tests were carried out on male and female combined data (M + F), female data alone (F) and male data alone (M), and were adjusted with ambient temperature (°C). The mean temperature (Mean Temp) ± one standard deviation (1SD) at which behavioural and metabolic tests were measured is given. Bold values denote significance. n_ID_, number of individuals; n_Obs_, number of observations.

### Collinearity of traits

Body size and body mass are frequently correlated. To check for collinearity of body size (pronotum width) and body mass, we performed Spearman’s rank correlations on female data alone and male data alone, using data from only those individuals from which RMR and AMR measures were obtained. We reasoned that collinearity of traits was present if the R_s_ value was equal to or higher than 0.7.

### Relationships between metabolic rate and exploratory behaviour/body mass/body size

To assess whether relationships between metabolic rate and body size/mass, and metabolic rate and exploratory behaviour exist across combined male and female data, we performed an LMM using RMR as the response variable. The LMM was performed on collated male and female data, and sex was included as a fixed term. The temperature at which measurements of metabolic rate were made (hereafter: metabolic temperatures) was included as a fixed term because metabolic rates are influenced by temperature (reviewed in: Schmidt-Nielsen, 2007). Pronotum width (as a proxy for body size) and body mass were also included as fixed terms. Temperatures impact also ectotherm behaviour (reviewed in: Abram et al., 2017) and may influence links between metabolism and behaviour (Hämäläinen et al., 2020). Exploratory behaviour interacting with the temperature at which exploratory behaviour was observed (hereafter: behavioural temperature) was, therefore, included as a fixed term. Personality traits have been shown to relate to morphological traits (*e.g.*, Kern et al., 2016). We therefore included exploratory behaviour as a fixed term interacting with body mass in our model. To account for changes in the temperature and light-dark conditions experienced by individuals over time, the week (week 1–10) in which beetles’ metabolism was measured, and their identity (‘ID’) were included as random terms. Removal of one outlier from the dataset did not qualitatively change the results (not presented).

We performed a generalised linear mixed model (GLMM) using AMR as the response variable. We used a gamma error structure with a log link in our GLMM to account for increased AMR variability with increasing exploratory behaviour, such that the AMR data were log-transformed. Fixed and random terms for the GLMM with AMR as response were as described for the LMM, however, as beetles were tested for their AMR only once, beetle ID was not included as a random term.

The sex-specificity of the effect of exploratory behaviour, body size and body mass on both RMR and AMR was determined by performing models as described above, on separate male and female datasets, with sex removed as an explanatory variable. Because male RMR was not repeatable, the effect of exploratory behaviour, body size and body mass on male RMR was not assessed.

### Model simplification

Stepwise model simplification was performed on LMMs and GLMMs; fixed terms were removed from these models in stages and compared to the previous model using likelihood ratio tests (Crawley, 2007). At each stage, the least significant fixed term, with the smallest effect on the model’s power was removed. All models were carried out using the lme4 package (Bates et al., 2015). Effects sizes of minimum adequate models were calculated using the MuMIn package (Barton, 2009).

### Ethics

The collection of beetles utilised in this study was carried out with a permit granted by the Lower Saxon State Department for Waterway, Coastal and Nature Conservation authorities (number: H72.2220212019).

## Results

Individual *C. hortensis* showed consistent differences in exploratory behaviour across 13–15 days, and in AMR across 4–8 h, for combined male and female data (hereafter: all beetles; Table 1) and each sex separately. RMR was repeatable over 13–15 days across all beetles and in females but not in males (Table 1). Body size (pronotum width) was significantly, positively correlated with body mass in males (Spearman rank correlation; R_s_ = 0.433, *p* = 0.005, *n* = 22), but not in females (R_s_ = 0.119, *p* = 0.323, *n* = 46). Average body size, body mass and their ranges are reported separately for males and females in Table S1.

**Figure 1:**
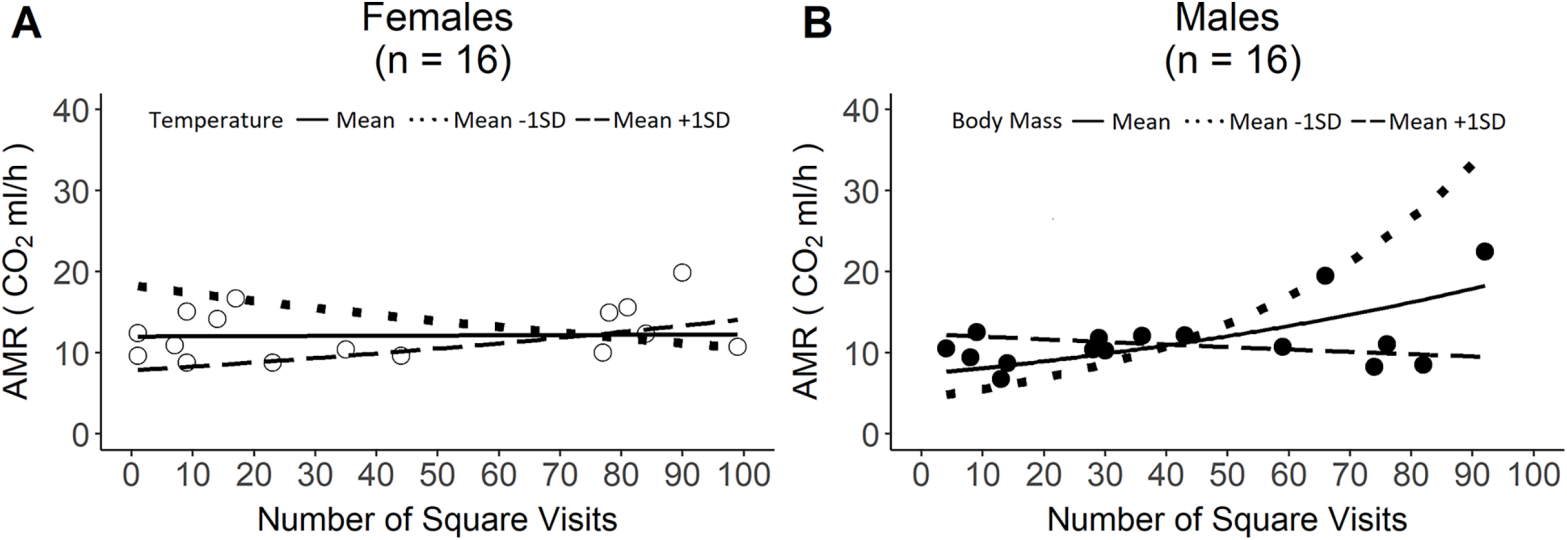
Sex-specific relationships between active metabolic rate (AMR) and exploratory behaviour. **(A) The relationship between AMR and exploratory behaviour at the mean temperature, mean +1 SD temperature, and mean −1 SD temperature in females (*n* = 16). (B) The relationship between AMR and exploratory behaviour at the mean body mass, mean +1 SD body mass, and mean −1 SD body mass, in males (*n* = 16).** Lines represent the predicted relationships from model outputs, back-transformed from a model with a log-link function.

More exploratory individuals had lower RMR for all beetles (Table S2), but there was no significant relationship between female RMR and exploratory behaviour (Table S2). AMR was significantly related to exploratory behaviour for all beetles (Table 2), and for females alone (Table 2, Fig. 1A). In both cases, the relationship between AMR and exploratory behaviour depended upon behavioural temperature. Male AMR was also significantly related to exploratory behaviour, however, this relationship depended upon body mass (Table 2, Fig. 1B).

**Table 2:** GLMMs for active metabolic rate (AMR) (CO_2_ ml/h) for males and female combined data (M + F), female data alone (F) and male data alone (M).

Dataset	Random Term	Var.	Fixed Term	Coeff.	χ^2^	DF	*p* value
M + F	Week	0.008	Intercept	3.05			
*n* = 32	Residual	0.054	B_Temp_: Expl.	<0.01	6.57	1	**0.010**
			Body Mass: Expl.	(−0.01)	1.40	1	0.237
			Body Mass	(−0.28)	0.24	1	0.623
			Expl.	−0.03			
			Pronotum Width	(0.04)	0.22	1	0.641
			M_Temp_	(0.08)	1.50	1	0.221
			B_Temp_	−0.03			
			Sex (M)	(−0.12)	1.73	1	0.189
F	Week	0.072	Intercept	2.67			
*n* = 16	Residual	0.018	B_Temp_: Expl.	<0.01	11.77	1	**<0.001**
			Body Mass: Expl.	(−0.07)	2.01	1	0.156
			Body Mass	1.42	4.74	1	**0.030**
			Expl.	−0.04			
			Pronotum Width	−0.37	10.71	1	**0.001**
			M_Temp_	0.21	6.52	1	**0.011**
			B_Temp_	−0.15			
M	Week	0.164	Intercept	4.77			
*n* = 16	Residual	0.005	B_Temp_: Expl.	(<0.01)	1.40	1	0.237
			Body Mass: Expl.	−0.19	41.93	1	**<0.001**
			Body Mass	7.84			
			Expl.	0.12			
			Pronotum Width	0.06	9.06	1	**0.003**
			M_Temp_	−0.24	18.81	1	**<0.001**
			B_Temp_	−0.12	15.10	1	**<0.001**

**Note:**

Data were log-transformed during analysis with the use of a log-link function. Coefficients (Coeff.) shown are not back-transformed. Behavioural temperature, B_Temp_; exploration (number of square visits in a novel environment), Expl.; metabolic temperature, M_Temp_; number of individuals, *n*; variance of random terms, Var. Coefficients (Coeff.) in square brackets belong to non-significant terms just before dropping those terms from the model. Bold *p* values denote significant terms.

**Figure 2:**
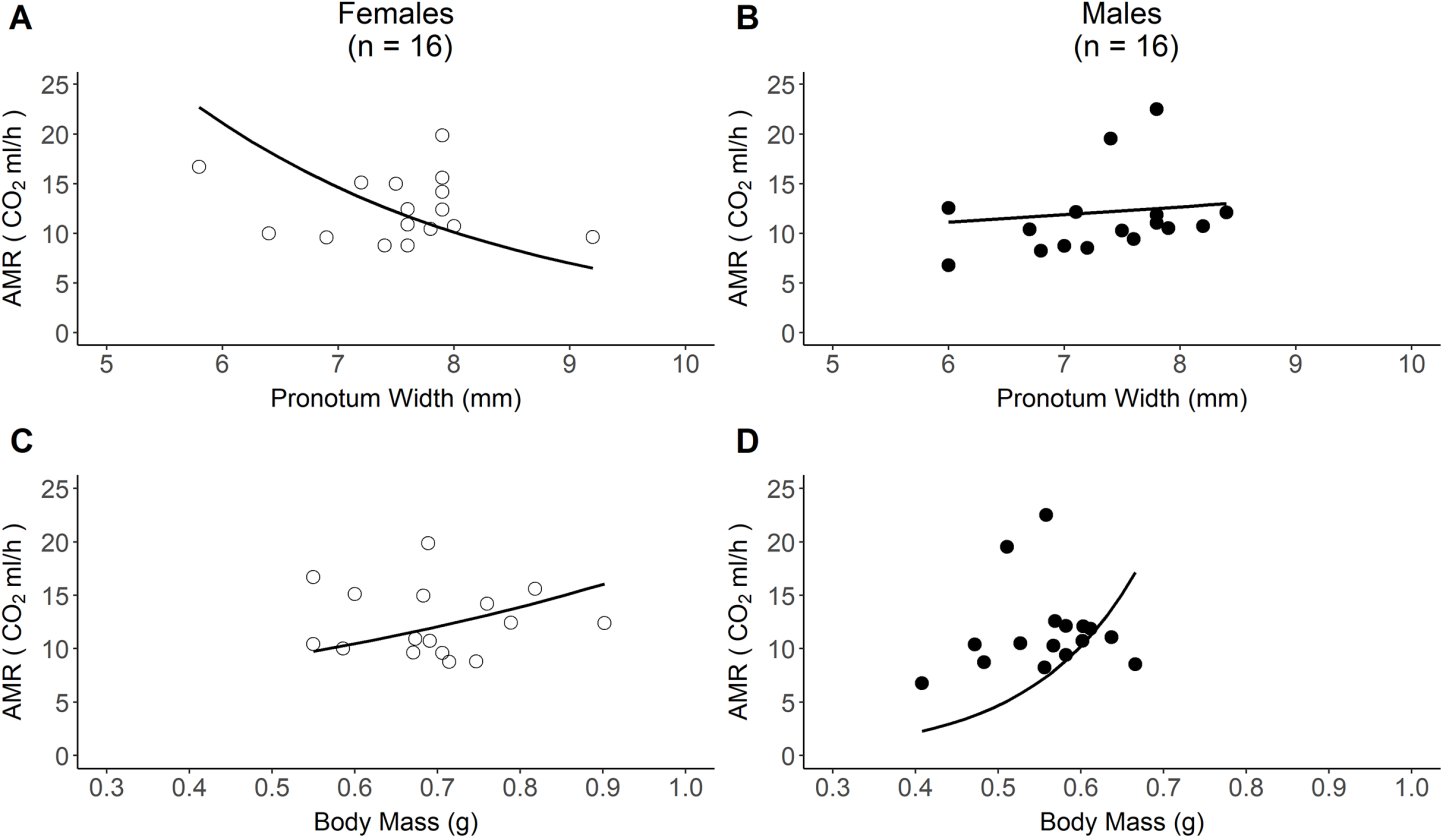
Sex-specific relationships between active metabolic rate (AMR) and both body size and body mass. **(A) The relationship between AMR and body size (measured as pronotum width) in females (*n* = 16). (B) The relationship between AMR and body size in males (*n* = 16). (C) The relationship between AMR and body mass in females (*n* = 16). (D) The relationship between AMR and body mass at average exploratory behaviour in males (*n* = 16).** Lines represent the predicted relationships from model outputs, back-transformed from a model with a log-link function.

AMR was unrelated to body size or mass for all beetles (Table 2). However, AMR did scale with both body mass and size in both males and females separately but did so differently between the sexes. Females with larger body sizes had significantly lower AMR (Table 2, Fig. 2A), whereas males with larger body sizes had significantly higher AMR (Table 2, Fig. 2B). Both male and female AMR increased with body mass: heavier females had significantly higher AMR (Table 2, Fig. 2C), as did heavier males with average exploratory behaviour (Table 2, Fig. 2D). However, per gram increase in body mass, the AMR of males that performed average exploratory behaviour (Table 2, Fig. 2D) increased more than female AMR (Table 2, Fig. 2C).

## Discussion

The relationships between metabolic rate and personality traits (*e.g.*, Biro et al., 2020; Cornwell, McCarthy & Biro, 2020), and between metabolic rate and morphology (*e.g.*, Baktoft et al., 2016; Bergstrom et al., 2019), have been studied in different taxa, but rarely in insects (but see: Royauté et al., 2015; Krams et al., 2017), or on a sex-specific basis (Hämäläinen et al., 2018), despite: (a) differences in physiology between insects and more commonly studied vertebrates (Schmidt-Nielsen, 2007); and (b) differences between males and females that might influence trait covariances (Hämäläinen et al., 2018). Consequently, our study is among the first to investigate sex-specific metabolic trait covariances with both a personality trait and morphology, in insects. *Carabus hortensis* AMR was related to body size/mass, though these relationships differed between sexes in terms of directionality for body size. Moreover, the relationship between AMR and exploratory behaviour depended upon behavioural temperature in females, but on body mass in males. Against our prediction, male RMR was not repeatable and hence its relationship with exploration or morphology was not assessed. Though repeatable, female RMR was unrelated to exploratory behaviour or body size/mass. Conversely, exploratory behaviour and AMR were repeatable in both sexes. Given the lack of female RMR trait covariances, and the lack of male RMR repeatability, we focus our discussion on AMR trait covariances.

The majority of studies of the relationships between metabolic rate, personality traits, body mass and size combine data from males and females (*e.g.*, Timonin et al., 2011) or analyse metabolic trait covariances in one sex alone (*e.g.*, Wells & Taigen, 1989; Royauté et al., 2015; White, Kells & Wilson, 2016). By comparing metabolic trait covariances in all beetles with that of males and females alone, we show that these relationships differed and that combining data for both sexes can lead to erroneous conclusions. This may help to explain why several studies across different taxa fail to find relationships between metabolic rate and personality traits/body mass (*e.g.*, Wells & Taigen, 1989; McDevitt & Speakman, 1996; Timonin et al., 2011). For example, the AMR-exploration relationship was temperature-dependent for all beetles but was body mass-dependent for males alone. Furthermore, AMR was unrelated to body mass when analysing all beetles but was significantly related to body mass when the sexes were considered separately. Such differences between sexes may arise from differences in reproductive strategies and investment as a consequence of anisogamy (Bateman, 1948; Maynard Smith, 1978; Hämäläinen et al., 2018).

In line with our predictions, differences in the relationship between AMR and exploratory behaviour occurred between sexes. The male AMR-exploration relationship was influenced by body mass, suggesting males of different weights have different proportions of metabolically active tissues. Conversely, the female AMR-exploration relationship was temperature-dependent. Such differences in the AMR-exploration relationship may arise from sex differences in activity and exploration related to reproduction. Males of other *Carabus* species are thought to search for females with whom to mate (*e.g.*, Drees & Huk, 2000), meaning that exploration or activity may influence the reproductive success of males more than that of females. Male *C. hortensis* exploratory behaviour may therefore remain relatively stable across the context of temperature in comparison to female exploratory behaviour.

Although some studies have previously shown sex-specific relationships between AMR and behaviours (*e.g.*, Niitepõld et al., 2011; Moschilla, Tomkins & Simmons, 2019; Methling et al., 2020), these behaviours were not tested repeatedly. Thus, to our knowledge, ours is the first to investigate the relationship between AMR and repeatable behaviour or personality traits on a sex-specific basis. As predicted, our results demonstrate that sex can be an important factor in the relationships between (active) metabolic rate and personality traits. We hypothesise that the extent to which sexes diverge in their metabolic rate-personality trait relationships depends on the strength of difference between male and female reproductive success or survival associated with the personality trait; the greater the difference in the association between a personality trait and fitness between the sexes, the greater potentially the divergence in the metabolic rate-personality trait relationships between males and females.

The relationships between *C. hortensis* AMR and both body size and mass differed between males and females. Males that had larger body sizes had greater AMR, whilst larger bodied females had lower AMR. Both male and female AMR increased with body mass, such that heavier individuals had higher AMR, yet the relationship was stronger in males than in females. Our findings are in line with both: (1) our prediction that relationships between metabolic rate and body mass should be stronger in males in than in females; and (2) findings of the only comparable insect-based study, in which the relationship between AMR and body mass was stronger in male eucalyptus-boring beetles (*Phoracantha semipunctata*) than in females (Rogowitz & Chappell, 2000). In contrast, studies on vertebrates have found no significant difference in the AMR scaling relationships between males and females (*e.g.*, Peterson, Walton & Bennett, 1998; Gifford, Clay & Peterman, 2013).

Sex differences in the relationships between AMR and body mass/size may be explained by sex differences in the proportions and benefits of metabolically active tissues. Evidence across taxa (*e.g.*, Streicher, Cox & Birchard, 2012; reviewed in: Glazier, 2005) indicates that mass dependence of metabolic rates changes with body composition. Males of other *Carabus* species seem to actively search for females with whom to mate (*e.g.*, Drees & Huk, 2000), which is likely an adaptation to increase mate searching capacity. If male *C. hortensis* are more active than females as we hypothesise, then males may invest heavily in musculature (*i.e.*, metabolically costly tissue) to sustain increased bouts of movement and to increase chances of locating a potential mate. In contrast, female *C. hortensis* are more likely to store energy as lipids (*i.e.*, tissues that are less metabolically costly) to fuel egg production (Turin et al., 2003). Female *C. hortensis* remain relatively inactive until hungry (Szyszko, Gryuntal & Schwerk, 2004), which may be an adaptation to retaining energy resources that should be allocated towards egg production. Heavier females, but not males, may store proportionally more lipids than lighter individuals, thus potentially explaining sex differences in AMR scaling. Our arguments would benefit from further investigation of sex-differences in body composition, as direct measures of body composition were not obtained in this study. Sex differences in AMR scaling could have also been explained by intraspecific variation in the proportions of eggs carried by heavier *versus* lighter females, however, it is highly unlikely that females in our study were carrying any eggs as our measures of body mass were recorded outside of the reproductive season. The negative relationship observed between female AMR and body size may be caused by a trade-off between the two: large bodies incur high metabolic costs, but may not be beneficial to female *C. hortensis* fecundity because in gravid carabids, the abdomen often becomes distended to accommodate large numbers of eggs (Goulet, 1976). Fecundity itself is thought to increase with metabolic rate in animals in general (Réale et al., 2010).

## Conclusions

We sought to explore the sex-specific relationships between metabolic rate (RMR and AMR), body size, body mass and exploratory behaviour in *Carabus hortensis* ground beetles. We found that males and females had different AMR trait associations: males with larger body sizes had higher AMR, while the opposite was true of females. Moreover, while the relationship between male AMR and exploratory behaviour was body mass-dependent, the relationship between female AMR and exploratory behaviour was temperature-dependent. Our results are suggestive of sexually antagonistic selection, meaning that individuals may be unable to reach their optimum trait expression and trait correlations and may suffer reduced fitness as a result. This may be especially true in cases where the direction of trait covariances differs between the sexes (Hämäläinen et al., 2018). Our results emphasise that sex plays an important role in intraspecific AMR trait covariances, and may help to explain why studies across many taxa fail to find relationships between metabolic rate and personality traits or body mass/size (Wells & Taigen, 1989; McDevitt & Speakman, 1996). Future studies of the relationships between metabolic rate, personality traits and body mass/size should therefore be careful to analyse data from males and females separately.

## Supplemental Information

10.7717/peerj.12455/supp-1 Supplemental Information 1 Mean female (*N* = 46) and male (*N* = 22) pronotum width (mm) and body mass (g), and the range of pronotum widths and body masses measured for each sex. Data includes only those individuals used in RMR LMM and AMR GLMM analysis.

10.7717/peerj.12455/supp-2 Supplemental Information 2 LMMs for resting metabolic rate (RMR) for male and female data combined (M + F) and female-only (F) data. Behavioural temperature, B_Temp_; exploration (number of square visits in a novel environment), Expl.; metabolic temperature, M_Temp_; Variance of random terms, Var; number of individuals, N. Coefficients (Coeff.) in square brackets belong to non-significant terms just before dropping those terms from the model. Bold *p*-values denote significant terms.

10.7717/peerj.12455/supp-3 Supplemental Information 3 Raw data including repeated measures for active metabolic rate (AMR), resting metabolic rate (RMR) and exploratory behaviour (number of square visits in a novel environment). The type of respiration performed (continuous (C) or discontinuous (D)) during AMR measurements (AMR_Type) and during RMR measurements (RMR_Type) by each individual in each trial, and the temperatures at which AMR (AMRTemp), RMR (RMRTemp), and exploration (ExplorationTemp) were measured are given in °C. NA denotes that data could not be obtained either because: (a) the individual was not measured for its AMR; (b) the beetle performed discontinuous respiration; or (c) the individual performed continuous respiration, but remained active throughout a RMR trial, or inactive throughout an AMR trial, meaning that a measure of RMR or AMR could not be obtained (denoted as C*). AMR and RMR are measured in CO_2_ ml/h. Individuals marked with an asterisk (*) died before a second exploration or RMR test could be made.

10.7717/peerj.12455/supp-4 Supplemental Information 4 Data used for LMMs. The week during which the individual was measured for its active metabolic rate (AMR), resting metabolic rate (RMR), and exploratory behaviour (Exploration; the number of square visits in a novel environment), is given. The beetle ID, sex, body mass (g), and body size (pronotum width, in mm) of each individual is given. ExplorationTemp and MetabolicTemp describe the temperatures at which exploratory behaviour and metabolic rate (MR, CO_2_ml/h) were measured. ‘Type_MR’ denotes whether RMR or AMR was measured. Data was used for analysis only when individuals demonstrated continuous respiration. RMR was analysed only when individuals remained at rest for a period of 3 min or longer, and AMR was analysed from periods when individuals were active, as described in the ‘Metabolic Rate Analysis’ Methods section.

## Additional Information and Declarations

### Field Study Permissions

The following information was supplied relating to field study approvals (*i.e*., approving body and any reference numbers): The collection of beetles used in this study was carried out with a permit granted by the Lower Saxon State Department for Waterway, Coastal and Nature Conservation authorities (number: H72.2220212019).

### Data Availability

The following information was supplied regarding data availability: The raw data are provided in Tables S3 and S4. Table S3 was used for statistical analysis of ‘Consistency of exploratory behaviour and metabolic rates over time’. Table S4 was used for statistical analysis of ‘Relationships between metabolic rate and exploratory behaviour/body mass/body size’.
