# Supplementary material for: Sex-specific covariance between metabolic rate, behaviour and morphology in the ground beetle Carabus hortensis
Source: PeerJ. 2021 Dec 15;9:e12455. doi: 10.7717/peerj.12455 (PMC8684319; doi:10.7717/peerj.12455)
Supplement: Supplemental Information 1 — Data includes only those individuals used in RMR LMM and AMR GLMM analysis. [file peerj-09-12455-s001.docx]

| ***Sex*** | ***Pronotum Width (mm)*** | | ***Body Mass (g)*** | |
| --- | --- | --- | --- | --- |
|  | ***Average ± SD*** | ***Range*** | ***Average ± SD*** | ***Range*** |
| Female | 7.5 ± 0.79 | 5.8 - 9.5 | 0.703 ± 0.10 | 0.481 - 0.910 |
| Male | 7.3 ± 0.73 | 5.8 - 8.4 | 0.558 ± 0.06 | 0.408 - 0.666 |

**Table S1. The mean, standard deviation (SD), and range of female (n = 46) and male (n = 22) pronotum width (mm) and body mass (g).** Data includes only those individuals used in analysis.
