# Supplementary material for: Sex-specific covariance between metabolic rate, behaviour and morphology in the ground beetle Carabus hortensis
Source: PeerJ. 2021 Dec 15;9:e12455. doi: 10.7717/peerj.12455 (PMC8684319; doi:10.7717/peerj.12455)
Supplement: Supplemental Information 2 — Behavioural temperature, BTemp; exploration (number of square visits in a novel environment), Expl.; metabolic temperature, MTemp; Variance of random terms, Var; number of individuals, N. Coefficients (Coeff.) in square brackets belong to non-significant terms just before dropping those terms from the model. Bold p-values denote significant terms. [file peerj-09-12455-s002.docx]

| ***Dataset*** | ***Random Term*** | ***Var.*** | ***Fixed Term*** | ***Coeff.*** | **χ^2^** | ***DF*** | ***p value*** |
| --- | --- | --- | --- | --- | --- | --- | --- |
| M + F | Week | 1.397 | Intercept | 5.92 |  |  |  |
| n = 58 | ID | 4.801 | B_Temp_: Expl. | [<0.01] | 0.40 | 1 | 0.529 |
|  | Residual | 3.231 | Body Mass: Expl. | [0.20] | 1.66 | 1 | 0.197 |
|  |  |  | Body Mass | [0.56] | 0.04 | 1 | 0.846 |
|  |  |  | Expl. | -0.04 | 7.87 | 1 | **0.005** |
|  |  |  | Pronotum Width | [-0.36] | 0.62 | 1 | 0.430 |
|  |  |  | M_Temp_ | [0.30] | 2.81 | 1 | 0.094 |
|  |  |  | B_Temp_ | [-0.10] | 0.23 | 1 | 0.632 |
|  |  |  | Sex (M) | [-0.38] | 0.26 | 1 | 0.611 |
| F | Week | 1.293 | Intercept | 5.19 |  |  |  |
| n = 41 | ID | 6.519 | B_Temp_ : Expl. | [<0.01] | 0.25 | 1 | 0.615 |
|  | Residual | 3.162 | Body Mass: Expl. | [0.08] | 0.16 | 1 | 0.692 |
|  |  |  | Body Mass | [-1.04] | 0.02 | 1 | 0.896 |
|  |  |  | Expl. | [-0.03] | 2.30 | 1 | 0.129 |
|  |  |  | Pronotum Width | [-0.72] | 1.51 | 1 | 0.220 |
|  |  |  | M_Temp_ | [0.30] | 2.07 | 1 | 0.150 |
|  |  |  | B_Temp_ | [-0.01] | 0.02 | 1 | 0.881 |

**Table S2. LMMs for resting metabolic rate (RMR) (CO_2_ ml/h) for male and female data combined (M + F) and female-only (F) data** B_Temp_, Behavioural temperature; Expl., exploration(number of square visits in a novel environment); M_Temp,_ metabolic temperature; n, number of individuals; Var, variance of random terms. Coefficients (Coeff.) in square brackets belong to non-significant terms just before dropping those terms from the model. Bold p-values denote significant terms.
